# *PmD479* is an Unutilized Gene for Powdery Mildew Resistance in Common Wheat

**DOI:** 10.1111/pbi.70704

**Published:** 2026-06-24

**Authors:** Heng Tang, Bo Lyu, Yang Yu, Liwen Wang, Xiaomei Liu, Xuan Zheng, Baiqiang Yan, Ziqi Li, Tingting Zhang, Xuefei Chen, Lin Wang, Jing Zhang, Qiaoling Luo, Xuancheng Hao, Xingfeng Li, Junhua Peng, Lynn Epstein, Fei Ni, Fei He, Jiajie Wu, Daolin Fu

**Affiliations:** ^1^ State Key Laboratory of Wheat Improvement, College of Agronomy Shandong Agricultural University Tai'an Shandong China; ^2^ School of Life Sciences Qilu Normal University Jinan China; ^3^ Laboratory of Advanced Breeding Technologies Institute of Genetics and Developmental Biology, Chinese Academy of Sciences Beijing China; ^4^ University of Chinese Academy of Sciences Beijing China; ^5^ Spring Valley Agriscience Co., Ltd. Jinan Shandong China; ^6^ Department of Plant Pathology University of California Davis California USA; ^7^ CAS‐JIC Centre of Excellence for Plant and Microbial Science (CEPAMS) Institute of Genetics and Developmental Biology, Chinese Academy of Sciences Beijing China

**Keywords:** broad‐spectrum resistance, powdery mildew, wild emmer wheat

Powdery mildew, caused by *Blumeria graminis* f. sp. *tritici* (*Bgt*), threatens global wheat production. Wild emmer wheat ( *Triticum turgidum* ssp. *dicoccoides*, DIC) is a progenitor of common wheat ( *T. aestivum* L.) and harbours abundant powdery mildew (*Pm*) resistance genes (Wulff and Liu 2025). Here, we mapped and cloned *PmD479*, an unutilized *Pm* gene on chromosome 2BL in the DIC accession ‘DIC479’ (Marais et al. 2005), and introduced *PmD479* into common wheat. Given its broad‐spectrum efficacy and current rarity in common wheat germplasm, *PmD479* is now a valuable resource for diversifying powdery mildew resistance in wheat.

DIC479 seedlings were resistant to 11 Chinese *Bgt* isolates (Table S1). In response to *Bgt* isolate E09, DIC479 produced reactive oxygen species (ROS) and exhibited localized necrosis (Figure 1A). DIC479 and Langdon (LDN, a *Pm*‐susceptible durum wheat, *T. turgidum ssp. durum* ) were used to develop an F_2_ population, which showed a 3:1 segregation (157 resistant vs. 47 susceptible; *χ* ^2^ = 0.418, *p* > 0.05). Among 83 randomly selected F_2:3_ families, 23 showed homozygous resistance, 39 segregated for *Pm* resistance and 21 showed homozygous susceptibility, fitting a 1:2:1 ratio (*χ* ^2^ = 0.397, *p* > 0.05) (Table S2). Thus, *PmD479* acts as a single dominant gene for *Pm* resistance.

**FIGURE 1:**
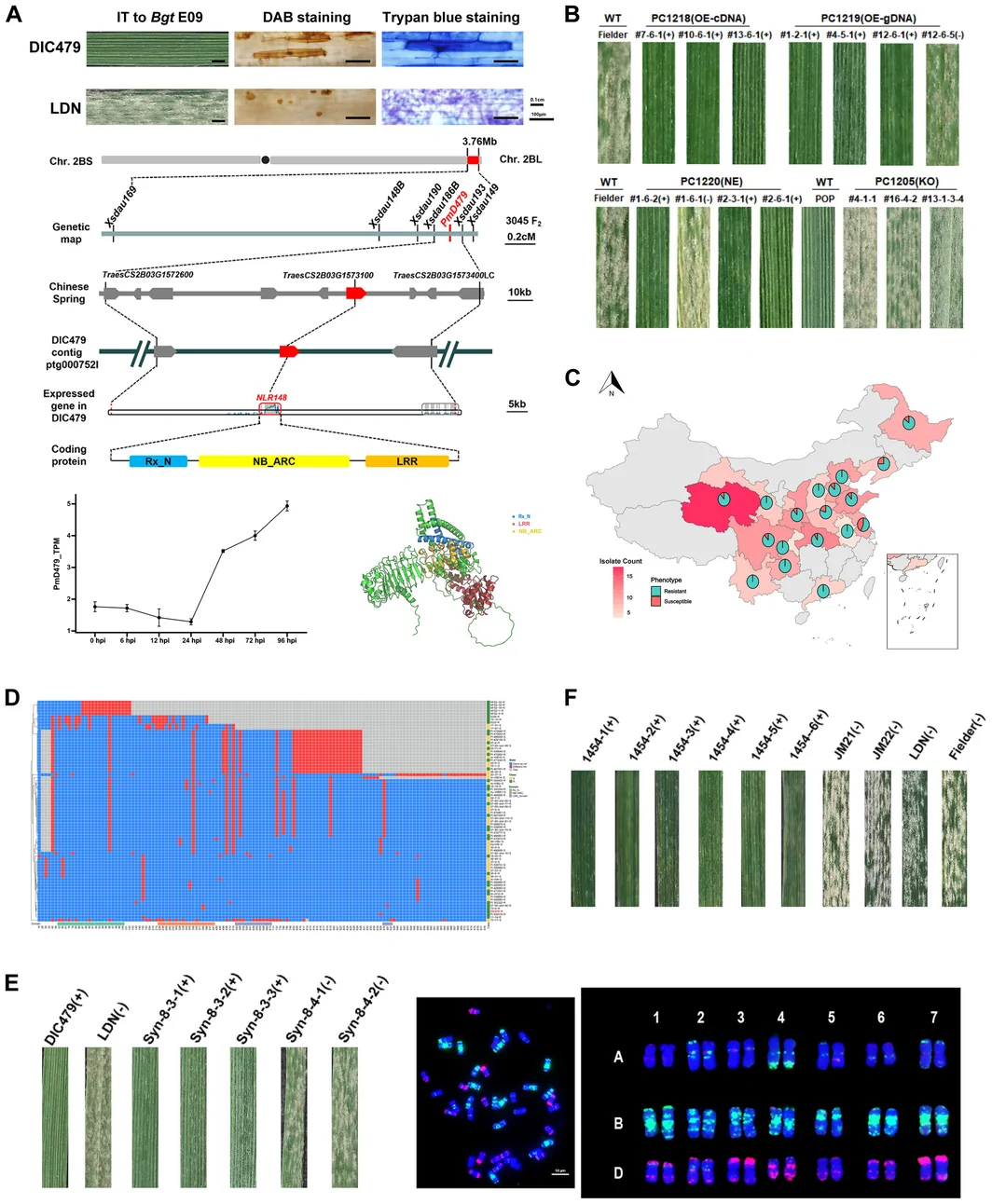
Cloning and functional validation of *PmD479*. (A) Symptoms and signs of *Bgt* on leaves of DIC479 and the susceptible LDN parents: Infection type (IT) with whitish mycelium on LDN; reactive oxygen species (ROS) with 3,3′‐diaminobenzidine (DAB) staining in DIC479; and dead cells in DIC479 and mycelium on LDN with trypan blue staining; Genetic and physical mapping of *PmD479*; the structure and expression of the candidate gene *NLR148*. (B) Responses to *Bgt* in T_1_ transgenic wheat leaves (PC1218 for *NLR148* cDNA overexpression; PC1219 for genomic DNA overexpression; PC1220 for native expression) and in *NLR148*‐edited wheat with PC1205. (C) The effectiveness of *PmD479* against 106 *Bgt* isolates. (D) The *PmD479* haplotype and *Pm* resistance among wild emmer wheat. Green: Resistant, yellow: Susceptible. Blue, red and grey indicate conserved, divergent or absent *PmD479*, respectively. (E) Syn‐8‐3 is *PmD479‐*positive, resistant to E09 and euploid (with 42 chromosomes in FISH). (F) The *Pm* phenotype in the *PmD479*‐positive ‘1454’ and the parents (JM21, JM22, LDN and Fielder). ‘(+)’ and ‘(−)’ denote presence or absence of *PmD479* in (B–F).

Nine *Pm*‐susceptible F_2_ plants were genotyped by the 90 K iSelect SNP array identified nine *PmD479*‐linked SNPs on the long arm of the homoeologous group 2 chromosome in wheat (Table S3). Sixteen molecular markers (Table S4) were developed by integrating the linked SNPs and the genomic collinearity among rice, barley, *Aegilops tauschii* and common wheat. Using 204 F_2_ plants, *PmD479* was mapped to a 2.35 cM interval between *Xsdau169* and *Xsdau149* on distal 2BL (Figure 1A).

To fine map *PmD479*, we sequenced DIC479 with PacBio HiFi, generated 8923 contigs with an N50 of 6.05 Mb, and developed 13 additional markers (Table S4). Using 124 homozygous recombinants from 3045 F_2:3_ plants, *PmD479* was precisely mapped between *Xsdau186B* and *Xsdau193* (< 0.1 cM; Figure 1A; Tables S4 and S5). Both *Xsdau186B* and *Xsdau193* reside on the ptg000752l contig (3.17 Mb), delimit a 90 kb region in DIC479, and correspond to 811.78–811.93 Mb (2BL) in the Chinese Spring reference genome v2.1 (Zhu et al. 2021) (Figure 1A). *NLR148*, a homologue of *TraesCS2B03G1573100*, is the only complete gene in contig ptg000752l (Figure 1A; Table S6). *NLR148* was constitutively expressed at 0 to 24 h post infestation (hpi) and then significantly upregulated at 48 to 96 hpi (Figure 1A). *NLR148* encodes for a typical resistance protein with 978 amino acids and three domains: a Rx N‐terminal domain (Rx_N), a NB‐ARC domain (NB‐ARC) and leucine‐rich repeats (LRR) (Figure 1A; Figure S1).

Using the *Pm*‐susceptible wheat ‘Fielder’ as the recipient, we transformed and expressed *NLR148* via three vectors: PC1218 (cDNA with maize ubiquitin promoter), PC1219 (genomic DNA with maize ubiquitin promoter) and PC1220 (native expression) (Figure S2). Transgenic plants, positive for either PC1218, PC1219 or PC1220, were highly resistant (infection types, ITs = 0) to E09, whereas non‐transgenic controls were highly susceptible (ITs = 4) (Figure 1B). For knockouts, we used CRISPR/Cas9 (PC1205) on a *Pm*‐resistant F_5_ line that was derived from a DIC479/LDN cross (Pop2‐167‐6). The knockouts from PC1205 resulted in either 1 or 15 bp deletions or a 1 bp insertion, and resulted in susceptibility to E09 (Figure 1B; Figure S3). Thus, *NLR148* is *PmD479* (GenBank: PX828566).

*PmD479* was further tested with 106 *Bgt* isolates from different areas in China. The Pop2‐167‐6 line was resistant to 93 isolates and immune or highly resistant to 78 of them (Figure 1C and Table S7). Thus, *PmD479* is effective against 88% of Chinese *Bgt* isolates, demonstrating broad‐spectrum resistance.

We surveyed the distribution and haplotype of *PmD479 in* 147 DIC accessions from the Fertile Crescent region. Homologous genes were PCR‐amplified from 75 accessions, revealing 28 haplotypes, with none identical to *PmD479* (Figure 1D; Table S8). In contrast, *PmD479* was absent in all 491 common wheat cultivars surveyed (Figure S4). Apparently, *PmD479* has not been deployed previously in common wheat.

Here, *PmD479* was then introduced into common wheat. A synthetic wheat, Syn‐8, was obtained between Pop3000‐2332‐17 (an F_5_ derivative of the DIC479/LDN cross) and *Aegilops tauschii* PI511383. The Pop3000‐2332‐17 was also crossed and backcrossed with common wheat cultivars and generated the hexaploid wheat 1454. Both Syn‐8‐3 and 1454 were euploid (42 chromosomes, confirmed by FISH; Figure 1E and Figure S5), positive for the *PmD479‐FM* marker, and resistant to E09 (Figure 1E,F; Tables S9 and S10). Therefore, *PmD479* introduces *Pm* resistance in common wheat and can be deployed in wheat breeding.

To date, 14 *Pm* genes have been mapped on chromosome 2BL in wheat. Twelve *Pm* genes, i.e., *Pm52/Pm6* (Qiu et al. 2025), *Pm33*, *Pm51*, *Pm63*, *Pm64*, *PmCG15‐009*, *PmKN0816*, *PmLS5082*, *pmQ*, *pmYN99102* (Wang et al. 2023) and *PmYD588* (Ma et al. 2021) (Table S11), differ from *PmD479* genetically and physically. However, the DIC‐derived resistance genes *MlZec1*, *MlAB10* and *PmD479* were localized to the 2BL deletion bin (0.89–1.00) (Mohler et al. 2005; Maxwell et al. 2010). Whether *PmD479* is allelic to *Mlzec1* and/or *MlAB10* remains unclear.

In summary, we cloned an unutilized *Pm* resistance gene *PmD479* from wild emmer wheat. Introduction of *PmD479* into common wheat markedly enhanced *Pm* resistance. Thus, this study provides a valuable gene and germplasm for powdery mildew resistance in common wheat.

## Funding

The Key R&D Program of Shandong Province, China (2024LZGC034, 2024CXPT072) and the Innovation Funding of the State Key Laboratory of Wheat Improvement (WIFY202519).

## Conflicts of Interest

The authors declare no conflicts of interest.

## Supporting information


Data S1.
Tables S1–S11.
Figures S1–S5.

## Data Availability

The sequencing data produced in this study have been deposited in the NCBI BioProject database under accession number PRJNA1394530.

## References

[pbi70704-bib-0001] Ma, P. , L. Wu , Y. Xu , et al. 2021. “Bulked Segregant RNA‐Seq Provides Distinctive Expression Profile Against Powdery Mildew in the Wheat Genotype YD588.” Frontiers in Plant Science 12: 764978. 10.3389/fpls.2021.764978 PMC8677838 34925412

[pbi70704-bib-0002] Marais, G. F. , Z. A. Pretorius , C. R. Wellings , B. McCallum , and A. S. Marais . 2005. “Leaf Rust and Stripe Rust Resistance Genes Transferred to Common Wheat From *Triticum dicoccoides* .” Euphytica 143: 115–123.

[pbi70704-bib-0003] Maxwell, J. J. , J. H. Lyerly , G. Srnic , et al. 2010. “ *MlAB10*: A *Triticum turgidum* Subsp. *Dicoccoides* Derived Powdery Mildew Resistance Gene Identified in Common Wheat.” Crop Science 50: 2261–2267.

[pbi70704-bib-0004] Mohler, V. , F. J. Zeller , G. Wenzel , and S. L. K. Hsam . 2005. “Chromosomal Location of Genes for Resistance to Powdery Mildew in Common Wheat ( *Triticum aestivum* L. Em. Thell.). 9. Gene *MlZec1* From the *Triticum dicoccoides* ‐Derived Wheat Line Zecoi‐1.” Euphytica 142: 161–167.

[pbi70704-bib-0005] Qiu, D. , L. Dong , C. Jiao , et al. 2025. “Wheat Powdery Mildew Resistance Gene *Pm52*/*Pm6* From *Triticum timopheevii* Encodes an NLR Protein With an Integrated PRK Domain.” Plant Communications 6: 101569. 10.1016/j.xplc.2025.101569 PMC12744728 41126526

[pbi70704-bib-0006] Wang, B. , T. Meng , B. Xiao , et al. 2023. “Fighting Wheat Powdery Mildew: From Genes to Fields.” Theoretical and Applied Genetics 136: 196. 10.1007/s00122-023-04445-4 37606731

[pbi70704-bib-0007] Wulff, B. B. H. , and Z. Liu . 2025. “Good Things Come in Pairs: Crop Disease Resistance From Sensor–Helper to Sensor–Executor Pairs.” Crop Journal 13: 1655–1659.

[pbi70704-bib-0008] Zhu, T. , L. Wang , H. Rimbert , et al. 2021. “Optical Maps Refine the Bread Wheat *Triticum aestivum* cv. Chinese Spring Genome Assembly.” Plant Journal 107: 303–314. 10.1111/tpj.15289 PMC8360199 33893684

