# Supplementary material for: PmD479 is an Unutilized Gene for Powdery Mildew Resistance in Common Wheat
Source: Plant Biotechnol J. 2026 Jun 24;24(10):5317–9. doi: 10.1111/pbi.70704 (PMC13398706; doi:10.1111/pbi.70704)
Supplement: Supplementary file 1 [file PBI-24-5317-s001.zip › pbi70704-sup-0001-Supinfo.docx]

**Materials and Methods**

**Plant materials**

*Triticum dicoccoide*s accession DIC479, collected from Israel, is resistant to powdery mildew, in contrast to the susceptible durum wheat cultivar Langdon (LDN). The highly susceptible common wheat Huixian Hong was employed as a control in disease assessments. To fine map *PmD479*, a mapping population comprising 3,249 F₂ plants was developed from a cross between DIC479 and LDN. Haplotype analysis was conducted on 147 wild emmer wheat accessions collected from the Fertile Crescent. Furthermore, Pop2-167-6-8 (an F₆ line derived from the DIC479 × LDN cross), homozygous for *PmD479*, was challenged with 106 isolates of *Blumeria graminis* f. sp. *tritici* (*Bgt*) collected from diverse regions across China. To investigate the distribution of *PmD479* in wheat, 491 common wheat accessions with publicly available genomic sequences were screened for the presence of this gene using datasets accessible at https://ngdc.cncb.ac.cn/gsa/search?searchTerm=CRA005878 and https://ngdc.cncb.ac.cn/gsa/browse/CRA004322.

To develop synthetic hexaploid wheat, the F_5_ plants derived from the DIC479×LDN cross, were genotyped using flanking markers for *PmD479* (*Xsdau148B* and *Xsdau149*) and for *QTug.sau-3B* (*Lsdau12* and *Xgpw1146*), a locus associated with unreduced gamete formation. A line homozygous at both loci, designated Pop3000-2332-17, was selected for hybridization with *Aegilops tauschii* accession PI511383. In parallel, to create germplasm introgressed with *PmD479*, Pop2-23-10 (an F_5_ plant from the same cross and homozygous for *PmD479*) was crossed and subsequently backcrossed with the powdery mildew susceptible common wheat cultivars Jimai 21, Fielder, and Jimai 22.

**Evaluation of powdery mildew resistance**

The powdery mildew resistance of DIC479 and Pop2-167-6-8 was evaluated by challenging them with 11 *Bgt* isolates obtained from the Institute of Plant Protection, Chinese Academy of Agricultural Sciences, and 106 *Bgt* isolates collected from various regions across China, respectively.

All infestations were performed in a controlled environment growth chamber or greenhouse under a 16 h light/8 h dark photoperiod at 24/20°C with approximately 70% relative humidity. Seedlings at the two-leaf stage were infested with the respective *Bgt* isolates. Infection types (ITs) were recorded at 7-10 days post-infestation (dpi) using a 0-4 scale, in which scores of 0-2 indicated resistance and scores of 3-4 indicated susceptibility. Each line was tested in two or three independent biological replicates to ensure phenotypic accuracy.

To assess reactive oxygen species (ROS) accumulation and cell death, seedlings were infested with *Bgt* isolate E09. Leaf samples collected at 7 days post-infestation were stained with 3,3'-diaminobenzidine (DAB) and Trypan blue according to previously described methods (Li et al. 2023). Bright-field images were captured using an Olympus BX51 optical microscope (Olympus, Tokyo, Japan).

**Genetic mapping**

For preliminary mapping of *PmD479*, 204 F₂ individuals from the DIC479 × LDN cross were genotyped using 16 molecular markers (Supplemental Table 4). These markers were developed based on the 90K iSelect SNP array (Wang et al. 2014) and reference genome sequences of rice, barley, *Aegilops tauschii*, and Chinese Spring. Initial mapping placed *PmD479* at the distal end of chromosome arm 2BL, flanked by markers *Xsdau169* and *Xsdau149*.

To refine the genetic interval, we screened 3,045 F₂ plants and their derived F₂:₃ families using the flanking markers *Xsdau169* and *Xsdau149*, and identified 124 homozygous recombinant lines. By combining the Chinese Spring reference genome (RefSeq v2.1, IWGSC 2018) with whole-genome sequencing data of DIC479, we developed 13 additional linked markers (Supplemental Table 4). These markers were used to genotype the homozygous recombinant lines for fine mapping *PmD479*. All primers were designed using Primer Premier 5 software.

**Genome assembly of DIC479**

High-molecular-weight genomic DNA was extracted from young leaves of DIC479. Genome sequencing was performed using the PacBio Revio system (Pacific Biosciences, CA, USA) with a REV-HiFi-20K library. In total, 6,739,432 HiFi reads were generated, yielding 128.67 Gb of sequencing data with a read N50 of 19,018 bp. De novo genome assembly was conducted using hifiasm v0.24.0 (Cheng et al. 2021) with default parameters.

**Gene expression analysis in the candidate interval**

Leaves of DIC479 were collected after *Bgt* isolate E09 infection. Total RNA was extracted using TRIzol reagent (Takara, Dalian, China) following the manufacturer’s instructions. RNA sequencing libraries were constructed using the NEBNext® Ultra™ RNA Library Prep Kit for Illumina (NEB, USA) and sequenced on an Illumina platform to generate 150 bp paired-end reads, yielding approximately 16 Gb of raw sequencing data.

Raw sequencing reads were quality filtered using fastp v0.21.0 (Chen et al. 2018). Clean reads were aligned to the assembled DIC479 genome using STAR v2.7.10b (Dobin et al. 2013). Duplicate reads were marked, and spliced alignments were processed using GATK v4.2.2.0 (McKenna et al. 2010) with the MarkDuplicates and SplitNCigarReads modules. Alignments with mapping quality ≥30 were extracted using samtools v1.13 (Li et al. 2009).

Read depth within the candidate genomic interval was calculated using a sliding-window approach with a window size of 200 bp and a step size of 50 bp. Coverage profiles were visualized using custom Python scripts.

**Transcriptome analysis of DIC479 at multiple time points post-infestation.**

Leaves of DIC479 were collected at 0 hour (h), 6h, 12h, 24h, 48h, 72h and 96h after E09 infection. Total RNA was extracted using TRIzol reagent (Takara, Dalian, China). For each sample, three independent biological replicates were prepared. RNA-seq libraries were constructed using the NEBNext® Ultra™ RNA Library Prep Kit for Illumina and sequenced on an Illumina platform to generate 150-bp paired-end reads, yielding no less than 10 Gb of raw sequencing data per sample.

Raw reads were filtered using fastp v0.21.0 (Chen et al. 2018) and aligned to the wild emmer wheat reference genome WEWSeq v1.0 (Avni et al. 2017) using STAR v2.7.10b (Dobin et al. 2013). Duplicate reads were marked, and spliced reads were processed using GATK v4.2.2.0 (McKenna et al. 2010). Gene-level read counts were generated using HTSeq (Anders, Pyl, and Huber 2015), and transcript abundance was quantified as TPM values using Salmon (Patro et al. 2017). Differentially expressed genes (DEGs) were identified using DESeq2 (Love, Huber, and Anders 2014) with default settings. Data visualization and graphical representation were performed using R and TBtools (Chen et al. 2020).

**The protein domains and three-dimensional model prediction for PmD479**

To elucidate the protein structural architecture of PmD479, we performed domain prediction for PmD479 using the SMART (http://smart.embl-heidelberg.de/) and InterPro (https://www.ebi.ac.uk/interpro/) databases. Subsequently, the tertiary structure of PmD479 was modelled utilizing AlphaFold3 (https://alphafoldserver.com/). The 'rank_0' model, exhibiting the highest confidence scores, was selected as the definitive predicted structure. Finally, the resulting three-dimensional model was visualized and manipulated using PyMOL 3.0.0 (https://pymol.org/). The image was rendered, labelled, and colored based on its distinct domains.

**Functional validation methods.**

To validate the function of *NLR148*, three overexpression vectors and one CRISPR/Cas9-based knockout (KO) vector were constructed for transgenic assays. The overexpression vectors PC1218 (Ubi::*NLR148* cDNA) and PC1219 (Ubi::*NLR148* gDNA) were driven by the maize (*Zea mays* L.) *ubiquitin* (*Ubi*) promoter. The natural-expression vector PC1220 (NP::*NLR148* gDNA::NT) contained the native promoter and terminator of *NLR148* to restore its endogenous expression pattern (Supplemental Figure 2). The genomic DNA (gDNA) sequence of *NLR148* is 3,315 bp, which includes a 2,937 bp coding sequence (CDS). In PC1220, the promoter region comprised 2,019 bp upstream of the start codon, and the terminator consisted of 1,070 bp downstream of the stop codon. Each vector (PC1218, PC1219, and PC1220) was independently transformed into *Agrobacterium* strain EHA105. Wheat transformation was performed via a *Agrobacterium*-mediated method (Ishida et al. 2015), using the powdery mildew-susceptible cultivar Fielder as the recipient.

The KO vector PC1205 contained two sgRNA sequences, 5’-GCACCCCCTCCGCAACCTTG-3’ and 5’-GTAATGCTGCCTCCAACTGC-3’, targeting 1032-1051 bp and 2302-2321 bp downstream of the start codon of *NLR148*, respectively. Pop2-167-6 (the F_5_ generation from the cross DIC479×LDN), carrying homozygous *NLR148* and resistance to E09, was used as the recipient.

The transgenic plants were grown in a growth chamber with 16 h light at 24℃ and 8 h darkness at 18℃. T_0_ plants of expression vectors were genotyped by amplifying *Bar* (*Bar*-F/R) and *PmD479* (*XML170*-F/R), while T_0_ plants of the KO vector were genotyped by amplifying *Bar* (*Bar*-F/R). Positive plants were subjected to Sanger sequencing.

**Haplotype analysis**

Based on the sequence of *PmD479*, two pairs of specific primers (*479Hap*-F1/*479Hap*-R1 and *479Hap*-F2/*479Hap*-R2) were designed. Genomic DNA was extracted from 147 accessions of wild emmer wheat. The target regions were amplified using these primers and subjected to Sanger sequencing. The resulting sequences were assembled to obtain *PmD479* alleles, and the corresponding amino acid sequences were deduced. Finally, a heatmap was generated with R (version 4.2.1) to visualize the haplotype patterns within this natural population.

**Cytogenetic analysis.**

Fluorescence *in situ* hybridization (FISH) was carried out as previously described (Du et al. 2017; He et al. 2017; Han et al. 2009). Germinating root tips were treated with nitrous oxide for 2 hours to arrest cell division and then fixed by immersion in 90% glacial acetic acid. The hybridization reaction volume was 10 µL per slide. FISH was performed using an oligonucleotide probe mixture containing 10 pmol each of AFA-3, AFA-4, pAs1-1, pAs1-4, pAs1-3, pAs1-6, and pSc119.2-1, plus 1 pmol of (GAA)_10_. For lines Syn-8-3 and 1454, AFA-3, AFA-4, pAs1-1, pAs1-4, pAs1-3, and pAs1-6 were 5’-end labeled with 5-TAMRA, while pSc119.2-1 and (GAA)_10_ were labeled with 5-FAM. All oligonucleotides were synthesized by Sangon Biotech (Shanghai, China). The FISH procedure followed He et al. (2017) with minor modifications. A 20 µL hybridization mixture containing probes, 50% formamide, 10% dextran sulfate, 2×SSC, and 0.1% SDS was denatured at 85 ℃ for 8 min, then applied to denatured chromosome slides and hybridized at 37℃ for 6 h. After rinsing with 2×SSC, slides were counterstained with 10 µL DAPI (Vector Laboratories). Images were captured using an Eclipse Ni-U fluorescence microscope and analyzed with NIS-Elements BR 4.00.12 software (NIKON). FISH analysis confirmed that Syn-8-3 and 1454 each carried 42 chromosomes with stable genetic backgrounds.

**The development of synthetic hexaploid wheat and introgression with *PmD479***

F_1_ plants were generated by crossing Pop3000-2332-17 (female) with *Aegilops tauschii* accession PI511383 (male), followed by embryo rescue at 14 days after pollination and natural chromosome doubling. These F1 plants were then selfed for two consecutive generations. Subsequently, synthetic hexaploid wheat lines with stable 42 chromosomes were identified through fluorescence *in situ* hybridization (FISH) and designated as Syn-8-3.

To enhance powdery mildew resistance in common wheat varieties, we crossed Pop2‑23‑10 (a homozygous *PmD479*‑carrying F_5_ line derived from DIC479×LDN, as the male parent) with the susceptible variety Jimai 21 (as the female parent) to obtain the F_1_ generation. The F_1_ plants were then used as the female parent to cross with two powdery mildew susceptible cultivars, Fielder and Jimai 22, successively, backcrossed consecutively for two generations, yielding the BC_2_F_1_ population. After two generations of selfing and molecular marker‑assisted selection (MAS) by *PmD479‑FM*, we selected the BC_2_F_3_ introgression line 1454, which carries homozygous *PmD479*.

**The development of functional markers**

Based on sequence polymorphisms at the *PmD479* locus among DIC479, LDN, Jimai 21, Fielder, and Jimai 22, a dominant functional marker, *PmD479-FM*, was developed. Bioassays and Sanger sequencing of synthetic hexaploid wheat and *PmD479* introgression lines confirmed the usefulness of the *PmD479-FM* marker for identifying germplasm with the *PmD479* introgression.

**Supplemental References**

Anders, S., Pyl, P.T., and Huber, W. 2015. HTSeq--a Python framework to work with high-throughput sequencing data. *Bioinformatics*, **31**, 166-169.

Avni, R., Nave, M., Barad, O., *et al*. 2017. Wild emmer genome architecture and diversity elucidate wheat evolution and domestication. *Science*, **357**, 93-97.

Chen, C., Chen, H., Zhang, Y., *et al.* 2020. TBtools: An Integrative Toolkit Developed for Interactive Analyses of Big Biological Data. *Molecular plant*, 13: 1194-202.

Chen, S., Zhou, Y., Chen, Y., and Gu, J. 2018. fastp: an ultra-fast all-in-one FASTQ preprocessor. *Bioinformatics*, **34**, i884-i890.

Cheng, H., Concepcion, G.T., Feng, X., *et al*. 2021. Haplotype-resolved de novo assembly using phased assembly graphs with hifiasm. *Nature Methods*, **18**, 170-175.

Dobin, A., Davis, C.A., Schlesinger, F., *et al*. 2013. STAR: ultrafast universal RNA-seq aligner. *Bioinformatics*, **29**, 15-21.

Du, P., Zhuang, L., Wang, Y., *et al*. 2017. Development of oligonucleotides and multiplex probes for quick and accurate identification of wheat and *Thinopyrum bessarabicum* chromosomes. *Genome*, **60**, 93-103.

Han, F., Gao, Z. and Birchler, J.A. 2009. Reactivation of an inactive centromere reveals epigenetic and structural components for centromere specification in maize. *The* *Plant Cell*, **21**, 1929-1939.

He, F., Xing, P., Bao, Y., *et al*. 2017. Chromosome Pairing in Hybrid Progeny between *Triticum aestivum* and *Elytrigia elongata*. *Frontiers in Plant Science*, **8**, 2161.

Ishida, Y., Tsunashima, M., Hiei, Y. and Komari, T. 2015. Wheat (*Triticum aestivum* L.) transformation using immature embryos. *Methods in molecular biology*, **1223**, 189-198.

Li, H., Handsaker, B., Wysoker, A., *et al*. 2009. The Sequence Alignment/Map format and SAMtools. Bioinformatics, **25**, 2078-2079.

Li, Y., Roychowdhury, R., Govta, L., *et al*. 2023. Intracellular Reactive Oxygen Species-Aided Localized Cell Death Contributing to Immune Responses Against Wheat Powdery Mildew Pathogen. *Phytopathology*, **113**, 884-892.

Love, M. I., Huber, W. and Anders, S. 2014. Moderated estimation of fold change and dispersion for RNA-seq data with DESeq2. Genome biology, **15**, 550.

McKenna, A., Hanna, M., Banks, E., *et al*. 2010. The Genome Analysis Toolkit: a MapReduce framework for analyzing next-generation DNA sequencing data, *Genome research*, **20**, 1297-1303.

Patro, R., Duggal, G., Love, M. I., Irizarry, R. A. and Kingsford, C. 2017. Salmon provides fast and bias-aware quantification of transcript expression. *Nature methods*, **14**, 417-419.

Wang, S., Wong, D., Forrest, K., *et al*. 2014. Characterization of polyploid wheat genomic diversity using a high-density 90,000 single nucleotide polymorphism array. *Plant biotechnology journal*, **12**, 787-796.
