# Supplementary material for: PmD479 is an Unutilized Gene for Powdery Mildew Resistance in Common Wheat
Source: Plant Biotechnol J. 2026 Jun 24;24(10):5317–9. doi: 10.1111/pbi.70704 (PMC13398706; doi:10.1111/pbi.70704)
Supplement: Supplementary file 1 [file PBI-24-5317-s001.zip › pbi70704-sup-0002-TableS1-S11-FigureS1-S5.docx]

**Table S1. Infection types（ITs）of DIC479 to different** ***Bgt* isolates（CK：Chancellor）**

| Cultivars | ***Bgt* isolates** | | | | | | | | | | |
| --- | --- | --- | --- | --- | --- | --- | --- | --- | --- | --- | --- |
|  | E09 | E30-(2) | E15 | E21 | E26 | E06 | E18 | E05 | E13 | E32 | E20 |
| DIC479 | 0^a^ | 0 | 0 | 0 | 0 | 0 | 0 | 0; | 0; | 0; | 0; |
| Chancellor | 4^b^ | 4 | 4 | 4 | 4 | 4 | 4 | 4 | 4 | 4 | 4 |

^a^ IT 0 and 0; are highly resistant. ^b^ IT 4 is highly susceptible.

**Table S2. Segregation ratios of *PmD479* in DIC479 × Langdon populations**

| Cross | Generation | Number of the F_2_  plants or F_2:3_ families | Observed ratio | | | Expected ratio | χ^2^ | *P* value |
| --- | --- | --- | --- | --- | --- | --- | --- | --- |
|  |  |  | HR^a^ | Seg | HS |  |  |  |
| DIC479/Langdon | F_2_ | 204 | 157 |  | 47 | 3:1 | 0.418 | 0.519 |
| DIC479/Langdon | F_2:3_ | 83 | 23 | 39 | 21 | 1:2:1 | 0.397 | 0.820 |

**^a^** HR: homozygous resistant, Seg: segregating (heterozygous resistant), and HS: homozygous susceptible.

χ^2^_0.05,1_ = 3.84; χ^2^_0.05,2_ = 5.99

**Table S3.** **The *PmD479*-linked SNP sites identified by** **90K SNP iSelect array analysis**

| SNP Name | Wheat Chromosome | Genotype of Langdon | Genotype of DIC479 | Genotype of 9 susceptible F_2_ plants |
| --- | --- | --- | --- | --- |
|  |  |  |  |  |
| *BobWhite_c7274_333* | 2L | A | B | A |
| *Excalibur_c56550_71* | 2L | A | B | A |
| *Excalibur_c7971_1573* | 2L | A | B | A |
| *CAP7_c16_300* | 2L | A | B | A |
| *RAC875_c27102_377* | 2L | A | B | A |
| *RAC875_c37540_583* | 2L | A | B | A^*^ |
| *IACX11305* | 2BL | A | B | A |
| *BobWhite_c21827_104* | 2BL | A | B | A |
| *Ra_c35731_1269* | 2AL | A | B | A |
| *IAV1535* | N/A | A | B | A |
| *Tdurum_contig9071_215* | N/A | A | B | A |
| *Kukri_c96357_134* | N/A | A | B | A^*^ |

^*^One plant out of 9 F_2_ plants analyzed was heterozygous for this SNP marker.

**Table S5. Genotyping results of key homozygous recombinants^a^**

|  | *Sdau*  *148B* | *Xsdau*  *185* | *Xsdau*  *189* | *Xsdau*  *190* | *Xsdau*  *186B* | *Xsdau*  *178* | ***PmD***  ***479*** | *Xsdau*  *183* | *Xsdau*  *184* | *Xsdau*  *182* | *Xsdau*  *193* | *Xsdau*  *194* | *Xsdau*  *191* | *Xsdau*  *188* | *Xsdau*  *187* | *Sdau*  *149* | Phenotype^b^ |
| --- | --- | --- | --- | --- | --- | --- | --- | --- | --- | --- | --- | --- | --- | --- | --- | --- | --- |
| DIB479 | A | A | A | A | A | A | A | A | A | A | A | A | A | A | A | A | R |
| Langdon | B | B | B | B | B | B | B | B | B | B | B | B | B | B | B | B | S |
| 2296-2 | B | B | B | B | A | A | A | A | A | A | A | A | A | A | A | A | R |
| 2296-4 | B | B | B | B | A | A | A | A | A | A | A | A | A | A | A | A | R |
| 1748-1 | B | B | B | B | B | A | A | A | A | A | A | A | A | A | A | A | R |
| 1748-2 | B | B | B | B | B | A | A | A | A | A | A | A | A | A | A | A | R |
| 2327-1 | B | B | B | B | B | B | B | B | B | B | A | A | A | A | A | A | S |
| 64-5 | A | A | A | A | B | B | B | B | B | B | B | B | B | B | B | B | S |
| 1830-1 | A | A | A | A | B | B | B | B | B | B | B | B | B | B | B | B | S |
| 2192-3 | A | A | A | A | A | B | B | B | B | B | B | B | B | B | B | B | S |
| 313-1 | A | A | A | A | A | A | A | A | A | A | B | B | B | B | B | B | R |
| 686-4 | A | A | A | A | A | A | A | A | A | A | B | B | B | B | B | B | R |
| 1404-3 | A | A | A | A | A | A | A | A | A | A | A | B | B | B | B | B | R |
| 1404-6 | A | A | A | A | A | A | A | A | A | A | A | B | B | B | B | B | R |
| 278-1 | A | A | A | A | A | A | A | A | A | A | A | A | A | B | B | B | R |
| 278-4 | A | A | A | A | A | A | A | A | A | A | A | A | A | B | B | B | R |

^a^ A indicates homozygous dominant and B indicates homozygous recessive.

^b^ R, resistance and S, susceptibility were assessed with *Bgt* isolate E09.

Note: Rows = plant lines, columns = markers.

Table S4. The markers used in this study

Table S6. Annotated genes in the corresponding candidate interval of the Chinese Spring genome

Table S7: The resistance of *PmD479* to 106 *Bgt* isolates collected from China

Table S8: Haplotype and resistance identification of *PmD479* in 147 WEW

Table S9: Identification of genotypes and resistance to *Bgt* isolate E09 in synthetic hexaploid wheat

Table S10: Identification of genotypes and resistance to *Bgt* isolate E09 of introgression lines with *PmD479*

Table S11: The *Pm* genes on 2BL chromosome.

**Figure S1. The structure of *PmD479.***

The full-length of *PmD479* is 3,315 bp and is comprised of two exons (1-2,850 bp and 3,229-3,315 bp) and one intron (2,851-3,228 bp). Exons are depicted as filled boxes (Exon 1 and Exon 2), and the intron is shown as a connecting gray line. The start codon (ATG) and stop codon (TGA) are indicated. The scale bar represents 0.2 kb (200 bp).

**Figure S2. Schematic diagrams of the T-DNA regions of the plant expression vectors PC1218, PC1219, and PC1220.**

**A.** PC1218 overexpression vector carrying the *NLR148* coding sequence (cDNA) driven by the maize *Ubi* promoter, with a Nos terminator. **B.** PC1219 overexpression vector carrying the *NLR148* genomic DNA (gDNA) driven by the maize *Ubi* promoter, with a Nos terminator. **C.** PC1220: natural-expression vector containing the *NLR148* genomic DNA (gDNA) flanked by its native 5'-UTR and 3'-UTR, driven by the native promoter (NP) and terminated by the native terminator (NT) of *NLR148*. All three vectors contain a selectable marker cassette consisting of the *Bar* gene for herbicide resistance (driven by the 35S promoter with a Nos terminator), and the *Hyg* gene for hygromycin resistance (driven by tandem 35S promoters with CaMV 3'-UTR). LB and RB indicate the left and right borders of the T-DNA, respectively.

(Abbreviations: Ubi, ubiquitin; Nos, nopaline synthase; 35S, cauliflower mosaic virus 35S promoter; CaMV, cauliflower mosaic virus; Hyg, hygromycin resistance gene; Bar, bialaphos resistance gene; LB, left border; RB, right border.)

**Figure S3. Sanger sequencing of CRISPR/Cas9-edited *NLR148* T_1_ plants.**

**A.** Schematic representation of *NLR148* and its CRISPR/Cas9-mediated target site. The PAM sequence is highlighted in red. The designed sgRNAs are reverse complements to the genomic DNA of *NLR148*. **B.** Mutations in transgenic T_1_ plants were detected using the specific primer pairs *479-KO* (Supplemental Tables 4). The above plants exhibit homozygous mutations in *NLR148*, including either 1-15 bp deletions or a 1 bp insertion, all of which disrupt gene function.

**Figure S4. Genotype heatmap of the *PmD479* physical region in 491 wheat lines.**

A total of 491 wheat lines from multiple geographic regions were analyzed using resequencing data from public databases. Variants within the physical region spanning the *PmD479* gene were extracted to generate a genotype heatmap. The blue bar on the right indicates the physical position of the *PmD479* coding region. The *PmD479* region shows high sequence diversity across the wheat diversity panel. No wheat line was found to carry a haplotype identical to the *PmD479* reference sequence.

**Figure S5. Fluorescence in situ hybridization (FISH) analysis of line 1454.**

Left side: probed with multiplex oligonucleotides, including AFA-3, AFA-4, pAs1-1, pAs1-4, pAs1-3, pAs1-6, pSc119.2-1 and (GAA)_10_. Right side: Oligonucleotide multiplex FISH ideogram of line 1454 illustrating the distribution patterns of the probes across the 42 chromosomes of the A, B, and D genomes. Scale bar: 10 µm.
